# First identification of NDM-4-producing *Escherichia coli* ST410 in China

**DOI:** 10.1038/emi.2016.117

**Published:** 2016-11-23

**Authors:** Shangshang Qin, Mengmeng Zhou, Qijing Zhang, Hengxun Tao, Yafei Ye, Huizhi Chen, Lijuan Xu, Hui Xu, Ping Wang, Xianju Feng

**Affiliations:** 1 School of Pharmaceutical Sciences, Zhengzhou University, Zhengzhou 450001, China; 2 Collaborative Innovation Center of New Drug Research and Safety Evaluation, Zhengzhou 450001, China; 3 Department of Veterinary Microbiology and Preventive Medicine, College of Veterinary Medicine, Iowa State University, Ames, IA 50011, USA; 4 College of Animal Science, Yangtze University, Jingzhou 434025, China; 5 Department of Clinical Laboratory, The First Affiliated Hospital of Zhengzhou University, Zhengzhou 450001, China

Dear Editor,

The worldwide dissemination of New Delhi metallo-β-lactamase 1 (NDM-1), an Ambler class B metallo-β-lactamase (MBL) conferring resistance to all β-lactams except monobactams, is of great concern for public health. NDM-4, which differs from NDM-1 by a single amino acid substitution (Met154Leu), was demonstrated to possess increased carbapenemase activity.^1^ As an infrequent *bla*_NDM_ allele, only several sporadic cases of infections due to NDM-4-producing *Escherichia coli* have been described in India, Austria, and Europe.^1, 2, 3^ At present, only one NDM-4 plasmid (pJEG027; GenBank accession NO KM400601) has been characterized.^4^ To understand the epidemiology and control the spread of antibiotic-resistant pathogens, we have been investigating carbapenem-resistant *Enterobacteriaceae* from human patients in China. Previously, we reported a high incidence (33.3%) and endemic spread of NDM-1-positive *Enterobacteriaceae* in Henan Province.^5^ Here we describe the first NDM-4-producing *E. coli* ST410 isolate, which, to our knowledge, is the first case in China, and report the characterization of the *bla*_NDM-4_ harboring IncX3 plasmid.

*E. coli* 14–55 was obtained from a blood culture of a 14-year-old girl hospitalized at The First Affiliated Hospital of Zhengzhou University, Zhengzhou city, Henan province, on 6 June 2014. The girl did not have a history of foreign travel. Susceptibility testing was initially performed by a Vitek 2 system (bioMérieux, Marcy l'Etoile, France), and Minimum Inhibitory Concentrations (MICs) were determined using the broth microdilution method and the agar dilution method (for fosfomycin) following the CLSI guidelines, and the MIC results were interpreted according to the CLSI breakpoints.^6^ The European Committee on Antimicrobial Susceptibility Testing breakpoints (available at http://www.eucast.org/clinical_breakpoints/) were used for colistin and tigecycline. *E. coli* 14–55 was resistant to all β-lactams, including imipenem and meropenem, fluoroquinolones, gentamicin and tetracycline, and was susceptible to amikacin, fosfomycin, tigecycline and colistin (Supplementary Table S1). The modified Hodge test and the imipenem-EDTA double-disk synergy test revealed that *E. coli* 14–55 was an MBL-producer.^6^ PCR and sequencing were performed to screen for the presence of *MBL* genes, including *bla*_IMP_, *bla*_NDM_, and *bla*_VIM_; only *bla*_NDM_ was detected.^5^ The complete coding sequence (CDS) of *bla*_NDM_ was amplified with a pair of primers (NDM-F: 5′-GAA TTC GCC CCA TAT TTT TGC; NDM-R: 5′-AAC GCC TCT GTC ACA TCG AAA T) that flanked the *bla*_NDM_ gene, and DNA sequencing revealed the presence of *bla*_NDM-4_ in *E. coli* 14–55. In addition, different *β-lactamase* encoding genes, including *bla*_CTX-M-15_, *bla*_SHV-12_, *bla*_TEM-1_ and *bla*_CMY-61_, together with the *qnrB* gene were identified in the NDM-4 positive isolate *E. coli* 14–55.^5^

*E. coli* phylogenetic group typing and multilocus sequence typing (MLST) showed that the isolate *E. coli* 14–55 belonged to phylogroup A and ST410.^5, 7^ At present, five *E. coli* STs (India: ST648; Italy and Denmark: ST405; Austria: ST167, ST4450, and ST101) have been identified carrying *bla*_NDM-4_ worldwide.^1, 2, 3^ Thus, this study represents the first report of ST410 as a carrier of *bla*_NDM-4_. Notably, recent publications from Germany and Italy described the clonal expansion of fluoroquinolone (FQ)-resistant CTX-M-15-producing *E. coli* ST410 in both human and animal populations.^8, 9^ Our previous and current studies also identified *E. coli* ST410 clinical isolates co-harboring multiple resistance determinants, including *bla*_NDM_ (*bla*_NDM-1/4_), ESBL_S_ (*bla*_CTX-M-15,_ *bla*_SHV-12_/*bla*_TEM-1_) and *ampC* genes (*bla*_CMY-30_/*bla*_CMY-61_).^5^ In addition, NDM-5 producing *E. coli* ST410 was recently detected in Egypt.^10^ These findings suggest that *E. coli* ST410 might be an emerging pandemic clone similar to ST131 for the dissemination of *CTX-M-15* or *NDM* encoding genes. This possibility remains to be examined from future surveillance work.

S1-PFGE and PCR-based plasmid replicon typing indicated *E. coli* 14–55 harbored three different plasmids that belonged to the IncX3, IncFIA and IncFIB incompatibility groups.^5, 11^ (Supplementary Figure S1). Southern hybridization using digoxigenin-labeled *bla*_NDM-1_-specific probes (Roche Applied Sciences, Germany) showed that *bla*_NDM-4_ was located on an approximately 50 kb plasmid in *E. coli* 14–55 (Supplementary Figure S1). Although the conjugal transfer of plasmids for *E. coli* 14–55 failed, the NDM-4 carrying plasmid belonging to the IncX3 incompatibility group was successfully transformed into *E. coli* DH5α Electro-Cells (TaKaRa, Dalian, China) by electroporation (Supplementary Figure S1). MICs for the transformant (named T55 in this study) are summarized in (Supplementary Table S1). To characterize the IncX3-type NDM plasmid, 12 pairs of primers were designed based on a reference IncX3 plasmid named pNDM-HN380 (NC_019162) for the mapping of the backbone regions (Supplementary Table S2), and the MDR regions were characterized by a primer walking method.

The *bla*_NDM-4_ harboring plasmid in *E. coli* 14–55 (named pEC55-NDM-4) is 54 035 bp in size, with an average G+C content of 49.1%. The sequence data revealed that pEC55-NDM-4 is highly similar (>99.99%) to plasmid pNDM-HN380, which is the first characterized *bla*_NDM-1_-harboring IncX3 plasmid of *Klebsiella pneumoniae* ST483 and was isolated in Hong Kong, with nucleotide changes from G to A at bp 4447, from T to G at 18 179 bp, from G to A at 21 656 bp, and from C to T at 23 844 bp (Figure 1).^12^ The point mutation at position T18,179 G corresponds to the A460C mutation in *bla*_NDM-1_, leading to the occurrence of the *bla*_NDM-4_ allele. A comparative plasmid analysis between pEC55-NDM-4 and pJEG027, which is the only completely sequenced NDM-4 plasmid of a *K. pneumoniae* strain isolated in Austria, provided support for Espedido *et al*'s hypothesis that pJEG027 might have arisen from a pNDM-HN380-like plasmid ancestor (for example, pEC55-NDM-4) through the events of a different IS*5* insertion and an IS*26*-mediated flanking deletion of *cutA1-groL*.^4^ Several recent studies revealed that the *bla*_NDM-4_ gene was carried by plasmids belonging to different replicon types, including F, FII, L/M and X3 in *E. coli* and *K. pneumoniae*; however, only two partial sequences of IncFII plasmids (accession NO KP826711 and KP826707) and one partial sequence of an IncX3 plasmid (accession NO KP826709) except pJEG027 are available for the genetic context of *bla*_NDM-4_.^2, 3, 13^ The second completely sequenced *bla*_NDM-4_ carrying plasmid pEC55-NDM-4, in our study, is nearly identical to the first characterized *bla*_NDM-1_-harboring IncX3 plasmid pNDM-HN380, further suggesting that *bla*_NDM-4_ might have emerged on IncX3 plasmids via a point mutation in *bla*_NDM-1_. The occurrence of a pNDM-HN380-like plasmid carrying *bla*_NDM-4,_ in this study, together with recent observations of IncX3-type plasmids carrying different *bla*_NDM_ alleles, including *bla*_NDM-1_ (pNDM-HN380), *bla*_NDM-4_ (pJEG027), *bla*_NDM-5_ (pNDM_MGR194, pNDM5_0215 and pEc1929), and *bla*_NDM-7_ (pEC50-NDM7), in different countries (Austria, India, China and Canada) and different species (*K. pneumoniae*, *E. coli*, *Serratia marcescens,* and *Enterobacter hormaechei*),^4, 12, 14, 15^ strongly indicates that IncX3 plasmids, which have a narrow host range in *Enterobacteriaceae*, might have played a major role in the rapid global dissemination of NDM-type MBLs among *Enterobacteriaceae*.

In summary, our study represents the first report of a NDM-4 producing *E. coli* isolate recovered from a blood culture of a patient without a history of foreign travel. The FQ-resistant *E. coli* 14–55 with multiple *β-lactamase* encoding genes, including *bla*_NDM-4_, *bla*_CTX-M-15_, *bla*_SHV-12_, *bla*_TEM-1_ and *bla*_CMY-61_, belonged to ST410, a potential international clone for the dissemination of CTX-M-15. Further surveillance is thus warranted to monitor the future dissemination of potentially endemic clones of ST410 that harbor *bla*_NDM_. The *bla*_NDM-4_ carrying IncX3 plasmid characterized in this study is nearly identical to pNDM-HN380 (with *bla*_NDM-1_), which was reported in Hong Kong. This finding, together with recent observations of IncX3 plasmids carrying different *bla*_NDM_ alleles in different countries, further suggests that IncX3 plasmids might have become a common vehicle for the dissemination of different NDM alleles among *Enterobacteriaceae* worldwide.

## Figures and Tables

**Figure 1:**
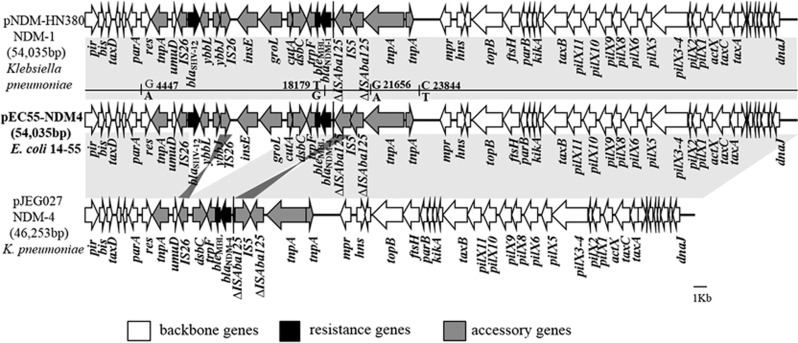
Comparison of linear plasmid maps of IncX3 plasmids harboring *bla*_NDM:_ pNDM-HN380 (*bla*_NDM-1_, JX104760), pEC55-NDM-4 (*bla*_NDM-4_, KX470734), pJE027 (*bla*_NDM-4,_ KM400601). Light gray shading indicates homologous regions, while dark gray shading indicates inversely displayed regions of homology. The different arrows indicate the positions, directions of transcription and predicted function of the genes.
