# Supplementary material for: First identification of NDM-4-producing Escherichia coli ST410 in China
Source: Emerg Microbes Infect. 2016 Nov 23;5(11):e118–. doi: 10.1038/emi.2016.117 (PMC5148021; doi:10.1038/emi.2016.117)
Supplement: Supplementary Information [file emi2016117x1.pdf]

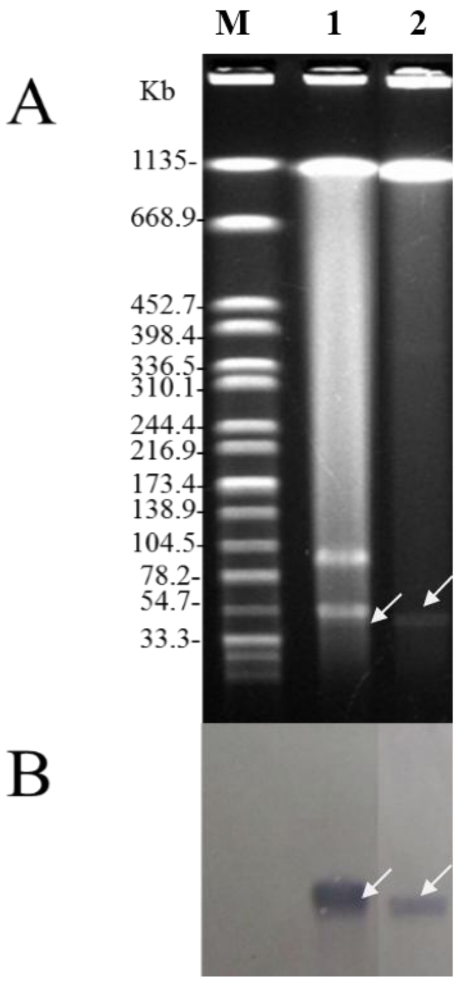

Supplementary Figure S1 Identification of *bla*$_{NDM}$ carrying plasmids. (**A**) Plasmid profiles revealed by S1 nuclease pulsed-field gel electrophoresis. (**B**) Southern blotting with *bla*$_{NDM-1}$ specific probe. Lane M, reference standard strain H9812 restricted with XbaI; Lane 1 and 2, *E coli* 14-55 and its transformant T55.
