# Supplementary material for: First identification of NDM-4-producing Escherichia coli ST410 in China
Source: Emerg Microbes Infect. 2016 Nov 23;5(11):e118–. doi: 10.1038/emi.2016.117 (PMC5148021; doi:10.1038/emi.2016.117)
Supplement: Supplementary Information [file emi2016117x2.pdf]

Supplementary Table S1 Antibiotic susceptibilities of *E.coli* 14-55, its transformant *E.coli* T55, and recipient strain *E.coli* DH5α.

Antimicrobial category	Antimicrobial agent	MIC (μg/mL)[a]		
		14-55[b]	T55[c]	DH5α
Penicillins	Ampicillin	>256	>256	≤2
Penicillins+β-lactamase inhibitors	Ampicillin/sulbactam	>256	>256	≤2
Antipseudomonal penicillins+β-lactamase inhibitors	Piperacillin/tazobactam	>256	>256	≤4
Non-extended spectrum cephalosporins	Cefazolin	>256	>256	≤4
Extended-spectrum cephalosporins	Ceftazidime	>32	>32	<0.06
	Cefepime	>256	>256	≤1
Carbapenems	Imipenem	>32	16	<0.06
	Meropenem	>32	16	<0.06
Monobactams	Aztreonam	>32	>32	<0.06
Fluoroquinolones	Levofloxacin	>32	≤0.25	<0.06
Aminoglycosides	Gentamicin	>32	0.5	1
	Amikacin	≤2	1	1
Folate pathway inhibitors	Trimethoprim/ sulfamethoxazole	≥320	≤20	≤20
Phenicols	Chloroamphenicol	4	2	1
Phosphonic acids	Fosfomycin	<1	<1	<1
Tetracyclines	Tetracycline	>32	2	1
Glycylcyclines	Tigecycline	≤0.5	≤0.5	≤0.5
Polymyxins	Colistin	0.5	0.5	0.5

[a] MICs were determined using the broth microdilution method and the agar dilution method (for fosfomycin) following the CLSI guidelines.
[b] *E.coli* 14-55 is positive for NDM-4.
[c] T55 is transformant of *E.coli* 14-55.
