# Supplementary material for: First identification of NDM-4-producing Escherichia coli ST410 in China
Source: Emerg Microbes Infect. 2016 Nov 23;5(11):e118–. doi: 10.1038/emi.2016.117 (PMC5148021; doi:10.1038/emi.2016.117)
Supplement: Supplementary Information [file emi2016117x3.pdf]

Supplementary Table S2 Primers used for PCR mapping of backbone of IncX3 plasmid in this study

**Primers**	**Location**	**PCR size (bp)**	**Nucleotide sequences (5'-3')**	**Reference**
X3MAP-F1	*res*		GCGTCATAGCGAGACTGATTAAC	this study
X3MAP-R1	upstream of *taxD*	3368	CAATGATGATCAGCCGTGAAG	
X3MAP-F2	upstream of *taxD*		GTGCGCTATGATTAACCGATG	this study
X3MAP-R2	*dnaJ*	3151	CCGGAAAGCGTATTCAGAACTT	
X3MAP-F3	*dnaJ*		TCCGGTGATCTGACTGAAAAGG	this study
X3MAP-R3	upstream of *taxA*	3065	GCAGTGAACTACCTCCACACAT	
X3MAP-F4	upstream of *taxA*		CGCTGAAGCACAATTTATGTGT	this study
X3MAP-R4	*pilX1*	3191	TGCATAAGACCGACTGAGTAACG	
X3MAP-F5	upstream of *pilX1*		TCTCGTGAGGAACTGGATAAAAT	this study
X3MAP-R5	*pilX3-pilX4*	3298	GTTGTCCACATCAAACGTATCGT	
X3MAP-F6	*pilX3-pilX4*		GCTCTTATCCGATCAGTAAGTTGC	this study
X3MAP-R6	downstream of *pilX6*	3036	CGGATACCGGAGGTTGTTTAT	
X3MAP-F7	*pilX6*		GCGTTAATATTGGCGCTAAGAG	this study
X3MAP-R7	*pilX10*	3093	CCAAATCCTGACCAACGATCAG	
X3MAP-F8	*pilX10*		ACCGACTATACAGCTAACAGTCG	this study
X3MAP-R8	*taxB*	3096	AATACGGGTCGAGGAAGTAGAG	
X3MAP-F9	*taxB*		GACAGGTTCAAGCAAGACAAGC	this study
X3MAP-R9	*ftsH*	3176	CCTTTATTTCAGCAGGAGTCGT	
X3MAP-F10	*ftsH*		GGCAGAATTGACAGAATCATATC	this study
X3MAP-R10	*hns*	3068	TTCGTCGATTGGTTTTGGTGT	
X3MAP-F11	*hns*		AAGAACGTCGTGAAGAAGCAG	this study
X3MAP-R11	downstream of *mpr*	3168	CCCCAATTCATCAATGAGCAC	
X3MAP-F12	downstream of *mpr*		ACAGTCATGGTCACGCAGTCTT	this study
X3MAP-R12	*tnpA*	3213	CGCAGACTTTCTTTCTCGTCG	
